# Mislocalization of Rieske Protein PetA Predominantly Accounts for the Aerobic Growth Defect of *tat* Mutants in *Shewanella oneidensis*

**DOI:** 10.1371/journal.pone.0062064

**Published:** 2013-04-11

**Authors:** Qixia Luo, Yangyang Dong, Haijiang Chen, Haichun Gao

**Affiliations:** Institute of Microbiology and College of Life Sciences, Zhejiang University, Hangzhou, Zhejiang, China; Arizona State University, United States of America

## Abstract

*Shewanella oneidensis* exhibits a remarkable versatility in respiration, which largely relies on its various respiratory pathways. Most of these pathways are composed of secretory terminal reductases and multiple associated electron transport proteins that contain cofactors such as Fe-S, molybdopterin, and NiFe. The majority of these cofactors are inserted enzymatically in the cytoplasm, and thus are substrates of the twin-arginine translocation (Tat) protein export system, which transports fully folded proteins. Using genomic array footprinting, we discovered that loss of TatA or TatC caused a reduction in the growth rate of *S. oneidensis* under aerobic conditions. Mutational analysis of the predicted Tat substrates revealed that PetA, the Rieske Fe-S subunit of the ubiquinol-cytochrome *c* reductase, predominantly dictates the aerobic growth defect of *tat* mutants in *S. oneidensis*. In addition, evidence is presented that the signal sequence in PetA appears to be resistant to cleavage after the protein is inserted into the cytoplasmic membrane.

## Introduction

In Gram-negative bacteria, there are now known six classes of protein secretion systems, currently identified as type I to type VI secretion system (TISS-T6SS), each of which shows a considerable diversity [1]. These systems, either individually or in combination, are responsible for translocation of more than one third of the proteome across, or inserted into the membrane [1]. A considerable portion of secretory proteins, which function in the periplasm, are substrates of the general secretion (Sec) and twin-arginine (Tat) pathways. Proteins that are secreted via the Sec pathway are targeted to a membrane-embedded Sec machinery by virtue of an N-terminal secretion signal and are subsequently translocated across the inner membrane in a linear unfolded conformation [2]. In contrast, the Tat pathway, identified first in the chloroplast thylakoid membrane and later in bacteria and archaea, is well known for its ability to export fully folded substrates out of the cytoplasm [3]–[5].

In Gram-negative bacteria, Gram-positive bacteria with high-GC-content genome, and plant chloroplast, the Tat pathway consists of three major membrane proteins, TatA, TatB, and TatC [3]. In contrast, most of Gram-positive bacteria possess a TatAC two-component system, in which the TatA subunit may be able to substitute for either TatA or TatB in *Escherichia coli* [3], [6]. The TatA and TatB proteins contain a single transmembrane spanning domain and the larger TatC protein possesses six transmembrane-spanning domains [3]. Some bacteria may possess additional proteins TatD and/or TatE [3]. It is uncertain whether TatD is a member of the Tat system as its previously defined role in a quality control system that recognizes malfolded Tat substrates and directs them to proteolysis has recently been disproven [7]. TatE is a low-expressed TatA homologue, which is largely dispensable in *E. coli* [1], [3]. The signature of Tat substrates is that their signal peptides contain a distinctive S/T-R-R-X-F-L-K (X is any polar amino acid) motif [3].

The importance of the Tat pathway varies substantially among organisms. The system is essential for viability of *Sinorhizobium meliloti* and *Bdellovibrio bacteriovorus* [8], [9]. More commonly, Tat pathway deficient mutants exhibit pleiotropic defects, including cell wall alterations, decrease in motility and in the ability to form biofilms, and increased susceptibility to antimicrobial agents [10]–[13]. In addition, an obvious growth defect has been reported in several bacteria, such as *Mycobacterium smegmatis*, *Ralstonia solanacearum* and *Staphylococcus carnosus*, but the underlying mechanism remains elusive [14]–[16]. Although the rationales for the use of the Tat pathway are not fully understood, a predominant portion of Tat substrates contain non-covalently bound cofactors [3]. For instance, the majority of *E. coli* 27 Tat substrates are cofactor-containing redox proteins, 13 of which have been experimentally confirmed [1], [3], [5], [17].

*Shewanella oneidensis*, a facultative Gram-negative anaerobe with great potential in bioremediation of metal contaminants in the environment, is renowned for its respiratory versatility [18]. To support this, the microorganism encodes a large number of terminal reductases and associated electron transport protein, such as up to 41 *c*-type cytochromes compared to 5–7 of *E. coli* [19], [20]. Virtually all of these proteins are either soluble periplasmic, attached to the periplasmic surface of the cytoplasmic membrane, or bound to the outer membrane. Moreover, a global profile of subcellular protein localization shows that more than one half of *S. oneidensis* proteome are exported to the periplasm or inserted into the inner or outer membrane [21]. Consistently, a genomic analysis of all sequenced *Shewanella* reveals that the genus encodes an exceptionally diverse set of secretion systems, including all of named types except for the type 4a secretion system (T4aSS) [22].

In this study, we utilized genomic array footprinting (GAF) to screen for genes that affect growth either negatively or positively in a high-throughput manner in *S. oneidensis* [23]. We discovered that the *tatA* and *tatC* mutants displayed impaired growth under aerobic conditions, a phenomenon that is rare in proteobacteria and has not been reported in γ-proteobacteria [1], [3], [5], [17]. Loss of the Tat pathway, however, failed to elicit any other defects that are commonly associated with the Tat system characterized to date. We then provided evidence that the deficiency of exporting a Tat substrate, PetA, the Rieske Fe-S subunit of ubiquinol-cytochrome *c* reductase, dictates the aerobic growth defect of *tat* mutants in *S. oneidensis*.

## Results

### Genetic array footprinting analysis of *S. oneidensis*

*S. oneidensis* has served as a model microbe for biogeochemistry studies for more than two decades and numerous reports on its anaerobic respiratory pathways have been published. In contrast, exploration into genes that play a crucial role in aerobic growth has been scarce although comparative genomics reveals plenty of surprises regarding this process [24]. Here we adopted a genomic array footprinting (GAF) technology for *S. oneidensis* and utilized it for identification of genes important for aerobic growth according to the method described elsewhere [23]. Plasmid pHGT214, derived from pBSL180 with the addition of a fragment containing a T7 promoter and a GGATCC site within the transposable element, was transferred into *S. oneidensis* for generation of a random mutant library [25]. A population of approximately 10^5^ independent mutants was pooled, aliquoted, saved, and used as the control sample (S_0_) when necessary. Three aliquots as biological replicates were subjected to competition experiments under aerobic conditions as described previously [26]. Cultures were sampled by centrifugation after 1 (S_1_) and 5 (S_5_) days, corresponding to 6.7 and 34 generations, respectively. The genomic DNAs were extracted, *in vitro* transcribed with T7 RNA polymerase, and dye-labeled by reverse transcription. To eliminate the interference of essential genes, these labeled cDNAs were co-hybridized with those generated from the control sample S_0_ onto the *S. oneidensis* whole genome microarrays and data were analyzed as previously described [27]–[29].

GAF analysis identified 96 genes that met the stringent selection criteria for the attenuated or enriched (Table S2). The attenuated outnumbered the enriched significantly, suggesting that gene interruption most often caused a reduction in the physiological fitness in competition against the wild type. Consistent with little knowledge about aerobic growth of *S. oneidensis*, a large number of genes whose loss introduced the substantially altered aerobic growth encode proteins whose function is currently unknown. Nevertheless, a number of genes encoding enzymes in energy metabolism were found to be significantly reduced in the S_5_ samples. Among them, AceE (pyruvate dehydrogenase complex, E1 component), CcoP (cytochrome *c* oxidase, *cbb*3-type, subunit III), Mdh (malate dehydrogenase), SdhA and SdhB (succinate dehydrogenase, flavoprotein subunit and iron-sulfur protein subunit, respectively), and SucC (succinyl-CoA synthase, beta subunit) are well characterized proteins that play roles in crucial biological processes to aerobic growth, such as the tricarboxylic acid cycle (TCA) and oxidative phosphorylation. The screen also identified some genes encoding elements of ABC transport systems, oligopeptide permeases, and *c*-type cytochromes although independent experimental validation is needed.

We were particularly intrigued in that both *tatA* and *tatC* were found to be attenuated, with less than 40% of that in the initial inocula remaining after 5 days. While this result indicates that the Tat pathway, as in γ-proteobacteria characterized thus far, is not essential in *S. oneidensis* [3], the Tat machinery of *S. oneidensis* is unlike its closely related counterparts in that it appears to be implicated in pathways critical to the bacterial aerobic growth. This is surprising because growth defect resulted from the removal of the system under aerobic conditions has yet been reported in this group of bacteria in which the Tat machinery is best understood [1], [3], [5], [17]. Conceivably, the Tat system transports some crucial proteins involved in aerobic respiration. Given that a large number of anaerobic terminal reductases and associate proteins may be transported via Tat pathway, this finding suggests that the system has key roles in both aerobic and anaerobic respiratory processes.

#### In silico analysis of the Tat system in S. oneidensis

**Figure 1:**
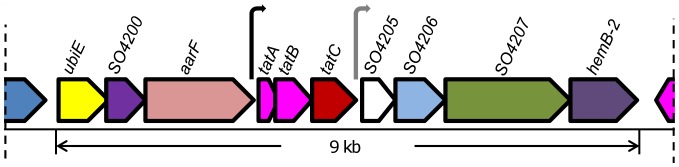
Genetic organization of *tat* genes from *S. oneidensis*. The marked region (∼9 kb) is a fragment perfectly conserved in all 25 sequenced *Shewanella* strains (img.jgi.doe.gov). Arrows represent predicted promoters, of which the one in black is conserved in all *Shewanella* and the other in gray is identified in 23 sequenced strains. *SO4206* is an analogue of *E. coli tatD*.

In all sequenced *Shewanella*, genes encoding Tat components reside on a perfectly conserved fragment (∼9 kb) in terms of ORFs although the first three genes, predicted to be involved in ubiquinone biosynthesis, appear to be unrelated to the Tat system (Fig. 1). The rest 7 genes on the fragment, namely *tatA*(*SO4202*), *tatB*(*SO4203*), *tatC*(*SO4204*), *SO4205*, *SO4206*, *SO4207*, and *hemB-2*(*SO4208*), are likely organized into two operons, one of which contains *tatABC*. TatA, TatB, and TatC of *S. oneidensis*, share 48%, 39%, and 61% identity in amino acid sequence with their *E. coli* counterparts respectively, with the highest homology observed in the N-terminus and predicted transmembrane domains. SO4206 appears to be an analogue of *E. coli* TatD based on 44% identity in amino acid sequence. However, given that the genome encodes two other proteins, SO1213 and SO2610, sharing sequence identities of ∼30% to *E. coli* TatD and distinct synteny compared to the *E. coli tat* cluster, whether SO4206 is the functional counterpart of *E. coli* TatD remains open. The operon containing *SO4206* encodes three other members. While SO4207 (GGDEF domain-containing protein) and HemB-2 (SO4208, delta-aminolevulinic acid dehydratase) is unlikely to be functionally related to the Tat system, little is known about SO4205, a periplasmic protein of 173 amino acids [30]. Additionally, the genome lacks a gene encoding a homologue of *E. coli* TatE, a redundant unit of TatA.

### Physiological characterization of *S. oneidensis* tat mutants

**Figure 2:**
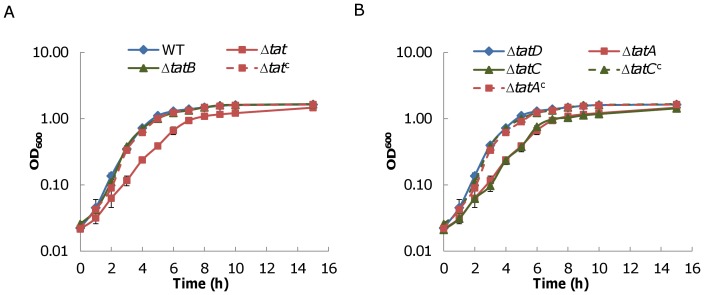
Growth of *S. oneidensis tat* mutants under aerobic conditions. Fresh M1 defined medium was inoculated with overnight cultures grown from a single colony by 1∶100 dilution, incubated on a shaker at 250 rpm at 30°C under aerobic conditions, and growth was measured by recording cell densities of cultures at 600 nm. For clarity, mutants were presented in two panels, A and B. Δ*tat* ^c^ represents Δ*tat* (Δ*tatABC*) containing complementation vector pHG101-*tatABC*. Δ*tatA* ^c^ and Δ*tatC* ^c^ represent Δ*tatA* and Δ*tatC* containing complementation vectors pHG101-*tatA* and pHG102-*tatC*, respectively. Growth assays were performed at least three times, with the error bars representing the standard deviation.

To obtain a comprehensive view of the *S. oneidensis* Tat system, a series of in-frame deletion strains were constructed and assayed for growth under aerobic conditions, including Δ*tatABC*, Δ*tatA*, Δ*tatB*, Δ*tatC*, and Δ*SO4206* (Fig. 2). As expected, the Δ*tatABC*, Δ*tatA*, and Δ*tatC* strains displayed significantly impaired growth when compared to the wild type. A quantitative analysis of growth revealed that generation times of these three mutants were approximately 75 min, more than two thirds over that (∼45 min) of the wild type. For all of these three mutants, genetic complementation restored the wild type growth rate, confirming that the observed growth defect was due to the corresponding mutations. In contrast, deletion of *tatB* and *SO4206* resulted in growth that was comparable to the wild type.

As Tat-deficient mutants display pleiotropic defects in general, we first assessed impacts of the *S. oneidensis* Tat system on the bacterial physiology by examining whether these mutant strains exhibit phenotypes established in other organisms. In *E. coli*, loss of the Tat system causes a distinctive chain-forming phenotype by mislocalization of amidases, which function to split two new daughter cells [31]. All of these mutants showed normal morphology, indistinguishable from the wild type, indicating that amidase(s) required for separation of new cells may not be a Tat substrate in *S. oneidensis* (Fig. S1A). This observation is supported by that the only periplasmic amidase (AmiB, SO0600) of *S. oneidensis* lacks a predicted Tat signal peptide. We then examined impacts of Tat on biofilm formation, motility, and resistance to SDS as these processes have been shown to be significantly affected by the loss of the Tat system in a variety of bacteria [3], [32], [33]. In all cases, the Δ*tatB* and Δ*SO4206* strains were unaffected (Fig. S1B, S1C and S1D). While deletion of *tatA*, *tatC*, and *tatABC* slowed phenotype development, none of the mutants was completely defective in any tested biological process when compared to the well-characterized non-motile strain Δ*fliD* and pellicle-deficient strain Δ*aggA* [34], [35]. More importantly, when differences in growth rates were considered all mutants were indistinguishable from the wild type, suggesting that proteins that are important in biofilm formation, motility, and susceptibility to antimicrobial agents may not be substrates of the *S. oneidensis* Tat system. Together with the aerobic growth defect observed in these mutants, these data implicate that the *S. oneidensis* Tat system may have a substantially different set of substrates.

### *S. oneidensis* Tat system recognizes *E. coli* Tat signal peptide

**Figure 3:**
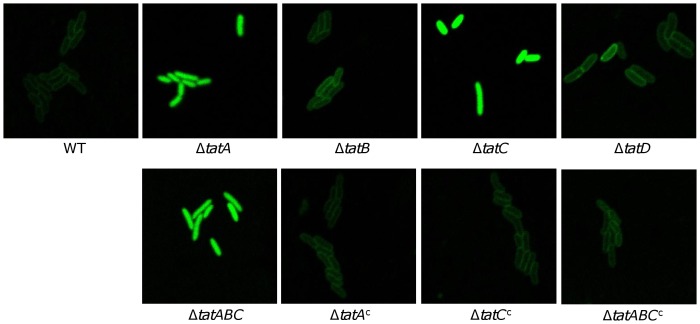
*S. oneidensis* Tat system recognizes *E. coli* Tat signal peptide. Fluorescence images of the wild type, *tat* mutant and their complemented strains carrying IPTG-inducible plasmid expressing hybrid protein TorA_Ec_-GFP (the signal peptide from the *E. coli* TorA fused to GFP). Cells at exponential phase (∼0.4 of OD_600_) were induced by 0.1 mM IPTG for 2 hours, and then sampled and visualized under a Zeiss LSM-510 confocal laser scanning microscope. The images were taken 1 h after the end of induction.

As the repertoires of *S. oneidensis* Tat substrates appear to be significantly different from those of the well-established Tat systems, we first examined whether the signal peptides of the *S. oneidensis* Tat substrates are altered. A GFP reporter whose transport is under control of the Tat signal peptide of *E. coli* TorA*_Ec_* (trimethylamine N-oxide reductase, a genuine Tat substrate in *E. coli*) was created and introduced into *S. oneidensis* strains for confocal microscopy observation [33] (Fig. 3). As in the *E. coli tat^+^* strain, specific targeting of GFP to the periplasm was observed for the *S. oneidensis* wild type strain. The same results were obtained with the *tatB* and *SO4206* mutant strains. On the contrary, in the Δ*tatABC*, Δ*tatA*, and Δ*tatC* strains the GFP fluorescence was evenly distributed in the cytoplasm, with no hint of enrichment of the signal at the periphery of the cells. Expression of the corresponding genes *in trans* in these strains reversed their defects in GFP transport. These data, collectively, conclude that the *S. oneidensis* Tat system is composed of two essential subunits TatA and TatC and is able to recognize *E. coli* Tat signal peptide. As loss of *tatABC*, *tatA*, and *tatC* results in identical phenotypes under all tested conditions, to simplify description we used the mutant devoid of the entire *tatABC* operon (Δ*tat*) for data presentation throughout the rest of this study although most of experiments were performed with all of these three mutants. It is worth mentioning that TatB of the *S. oneidensis* Tat system is surprisingly dispensable for function in contrast to all of the TatABC systems characterized to date [3]. The underlying mechanism will be described as part of a separate report.

### Substrates of Tat pathway in *S. oneidensis*

**Figure 4:**
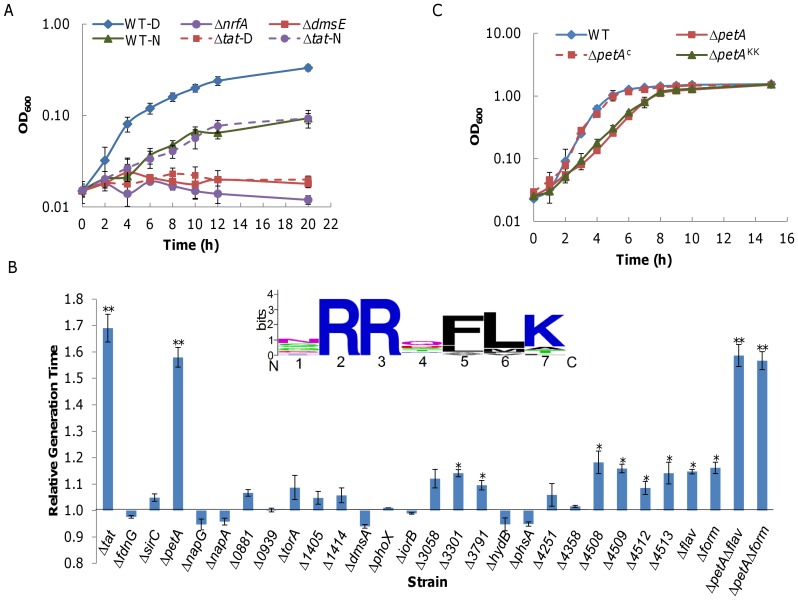
Characterization of mutants lacking one of Tat substrates in *S. oneidensis*. **A.** Growth of the wild type, Δ*tat*, Δ*nrfA*, and Δ*dmsE* with DMSO (D) or nitrite (N) as the sole electron acceptor under anaerobic conditions. **B.** Growth of *S. oneidensis* mutants lacking one of Tat substrates. Generation time of each strain was deduced from the early exponential phase (∼0.1 to 0.4 of OD_600_) and normalized to that of the wild type. Generation time of wild type is defined as 1.00. The single asterisk (*p*<0.05) and double asterisk (*p*<0.01) indicate significant difference in growth rate when compared to the wild type. The consensus motif in signal peptides of the 24 predicted Tat substrates prepared with WEBLOGO version 2.8.2 (http://weblogo.berkeley.edu) was shown. **C.** Growth of the wild type, Δ*tat*, Δ*petA*, and Δ*petA* complemented by pHG101 containing the wild type *petA* (Δ*petA* ^c^) or mutant *petA* (Δ*petA* ^KK^) under aerobic conditions. *petA* ^KK^ encodes a protein whose conserved RR within the consensus motif of signal peptides was replaced by KK. Growth assays were performed at least three times, with the error bars representing the standard deviation.

The finding that loss of Tat pathway causes growth defect is not new. It is conceivable that mislocalization of at least one of the Tat substrates leads to the impaired growth. As a first step to identify the protein(s) of interest in *S. oneidensis*, we predicted Tat substrates using programs TatFind, PRED-TAT and TatP on the *S. oneidensis* proteome for N-terminal Tat-targeting sequences [36]–[38]. In total, 29, 42, and 49 proteins were predicted to be Tat substrates by TatFind, PRED-TAT, and TatP (substrates with four “Yes” in five reference points taken as the identified), respectively (Table S3). Notably, all substrates identified by TatFind were included in those identified by PRED-TAT, suggesting that TatFind has a more stringent criterion. By contrast, the repertoire of substrates by TatP includes up to 30 members that neither TatFind nor PRED-TAT revealed, indicating that the program differs from the other two significantly. To assess the reliability of these predicted data sets, we tested Δ*tat* for its ability to utilize dimethyl sulfoxide (DMSO), and nitrite [26], [39], [40]. Under anaerobic conditions, Δ*tat* was compared to each terminal reductase-deficient mutant when the corresponding chemical was used as the sole electron acceptor (Fig. 4A). The *tat* mutant was able to respire on nitrite but not on DMSO, confirming that DmsA rather than NrfA, as predicted, is a substrate of Tat pathway.

To refine the Tat substrates, all candidates were subjected to membrane protein prediction and functional analysis based on genome annotation and sequence comparison. The resulting dataset containing 24 potential Tat substrates of *S. oneidensis* is given in Table 1. While the consensus motif in signal peptides of these 24 substrates highly resembled the canonical motif (S/T-R-R-X-F-L-K), it varied significantly at the first residue (Fig. 4B). We then constructed mutants in which one of these candidate genes was deleted and assayed their growth in comparison with the wild type and Δ*tat* strains. While most of deletion strains resulted in growth that was comparable to that of the wild type, seven displayed statistically significant growth defect of varying degrees (Fig. 4B). Genes whose removal resulted in a moderate decrease in growth rate belonged to two groups, flavocytochrome *c* (*SO1414*, *SO3058*, and *SO3301*) and formate dehydrogenase (*SO4508*, *SO4509*, *SO4512*, and *SO4513*). Interestingly, deletion of all three flavocytochrome *c* genes (Δ*SO1414*Δ*SO3058*Δ*SO3301*, Δ*flav* in Fig. 4B) or all four genes encoding formate dehydrogenase proteins (Δ*SO4508*Δ*SO4509*Δ*SO4512*Δ*SO4513*, Δ*form* in Fig. 4B) did not further impede growth (Fig. 4B). This may be explained by the fact that the *S. oneidensis* genome encodes multiple copies of these proteins [40]. The strain lacking *petA*, whose product is the Rieske Fe-S subunit of a ubiquinol-cytochrome *c* reductase, showed most significant reduction in growth rate and a copy of *petA* expressed *in trans* reversed the defect of the mutant (Fig. 4C). However, a statistical analysis of growth difference between the Δ*tat* and Δ*petA* strains revealed that the Δ*tat* strain had a slower growth rate than the Δ*petA* strain (*p*<0.05), implicating that there must be other substrates also contributory for growth defect of the Tat deficient strain. These substrates are unlikely to be flavocytochrome *c* or formate dehydrogenase because the additional removal of the *petA* gene from the Δ*flav* and Δ*form* strains resulted in growth that was identical to that of the Δ*petA* strain. Consistent with its role in transporting electrons to oxidases, deletion of *petA* did not affect anaerobic growth of *S. oneidensis* using either fumarate or DMSO as EA (data not shown). All together, the data from the mutational analysis of the predicted Tat substrates suggest that PetA is the most crucial substrate for growth defect of the Tat-deficient mutant and others may contribute.

**Table 1:** Refined putative Tat substrates in *S. oneidensis*.

Locus	Gene	Product	Predicted localization^a^
SO0101	*fdnG*	nitrate-inducible formate dehydrogenase, molybdopterin-binding subunit	P
SO0483	*sirC*	thiosulfate-inducible NrfC-like 4Fe-4S ferredoxin	OM
SO0608	*petA*	ubiquinol-cytochrome c reductase, FeS subunit, PetA	IM
SO0847	*napG*	periplasmic nitrate reductase, ferredoxin component, NapG	IM
SO0848	*napA*	periplasmic nitrate reductase, molybdopterin-binding subunit, NapA	P
SO0881		expressed periplasmic protein	P
SO0939		split-soret diheme cytochrome c	E
SO1232	*torA*	trimethylamine-N-oxide reductase, TorA	P
SO1405		transglutaminase family protein	P
SO1414		flavocytochrome c flavin subunit, putative	P
SO1429	*dmsA*	dimethyl sulfoxide reductase, molybdopterin-binding subunit, DmsA	OM
SO2385	*phoX*	monomeric alkaline phosphatase, PhoX	E
SO3048	*iorB*	isoquinoline 1-oxidoreductase, molybdopterin-binding subunit, IorB	P
SO3058		flavocytochrome c, flavin subunit	P
SO3301		flavocytochrome c, flavin subunit	IM
SO3791		peptidase, M19 family	P
SO3921	*hydB*	periplasmic [Fe-Fe] hydrogenase, small subunit, HydB	P
SO4062	*phsA*	sulfur reductase, molybdoperterin-binding subunit, PhsA	P
SO4151		secreted polysaccharide deacetylase	OM
SO4358	*dmaA-2*	outer membrane oxidoreductase, molybdopterin-binding subunit	OM
SO4508		formate dehydrogenase accessory protein	P
SO4509		formate dehydrogenase, molybdopterin-binding subunit, FdhA_1	P
SO4512		formate dehydrogenase accessory protein	P
SO4513		formate dehydrogenase, molybdopterin-binding subunit, FdhA_2	P

a P: periplasmic protein; OM: outer membrane protein; IM: inner membrane protein; E: extracellular protein.

### PetA is a Tat substrate

PetA, together with PetB (cytochrome *b* subunit of ubiquinol-cytochrome *c* reductase) and PetC (cytochrome *c*_1_ subunit of ubiquinol-cytochrome *c* reductase), forms the cytochrome *bc*_1_ complex, which transports electrons to the cytochrome *c* terminal oxidase. The observed phenotype of the Δ*petA* strain indicates that the lack of the cytochrome *bc*_1_ complex impairs aerobic growth, which is further supported by the finding that a *petC* mutant was defective to similar extent in aerobic growth [20].

Based on TMHMM prediction, *S. oneidensis* PetA has a transmembrane domain, which is consistent with the topology of its analogues in many other bacteria [41]. The N-terminal of the protein contains a short stretch of G-R-R-R-F-L-T, resembling the canonical motif X-R-R-X-F-L-K for the Tat translocon (Fig. 4B). It has been firmly established that invariant arginine residues (the underlined in the case of PetA) within the consensus motifs are critically important for the Tat transport as conservative substitution of either one by a lysine efficiently blocks the export of Tat substrates [42], [43]. To assess the essentiality of the Tat system for export of PetA, we replaced both signature arginines with lysines to produce a mutant PetA (PetA^KK^) and assayed growth of the Δ*petA* strain carrying PetA^KK^. As shown in Fig. 4A, expression of the mutant *petA* was no longer able to recover the growth defect, supporting that PetA is the substrate of the Tat system in *S. oneidensis*.

**Figure 5:**
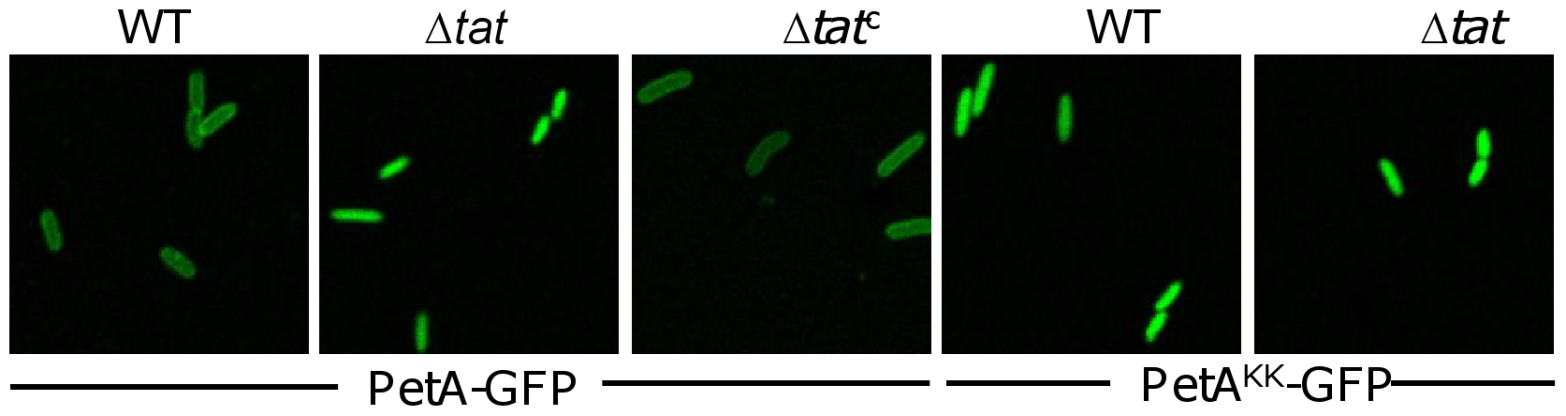
PetA is a Tat substrate in *S. oneidensis*. Fluorescence images of the wild type strain, Δ*tat* and its complemented strains carrying IPTG-inducible plasmid expressing hybrid protein PetA-GFP or PetA^KK^-GFP. Cells at exponential phase (∼0.4 of OD_600_) were induced by 0.1 mM IPTG for 2 hours, and then sampled and visualized under a Zeiss LSM-510 confocal laser scanning microscope. The images were taken 1 h after the end of induction.

To conclude that PetA is indeed a Tat substrate, the GFP was fused to the predicted signal peptide of PetA and expressed in the wild type and *tat* mutant strains. As shown in Fig. 5, in the wild type strain GFP fusions displayed a uniform peripheral localization pattern. In contrast, PetA-GFP lost their peripheral distribution in the Δ*tat* strain. The even-distributing phenotype resulting from the *tat* deletion was corrected by its expression *in trans*. More importantly, the even-distribution of PetA^KK^-GFP was independent of the Tat system, confirming that the Tat system is essential for the transport of PetA.

### Cleavage of Tat signal peptides is independent of Tat-mediated translocation

**Figure 6:**
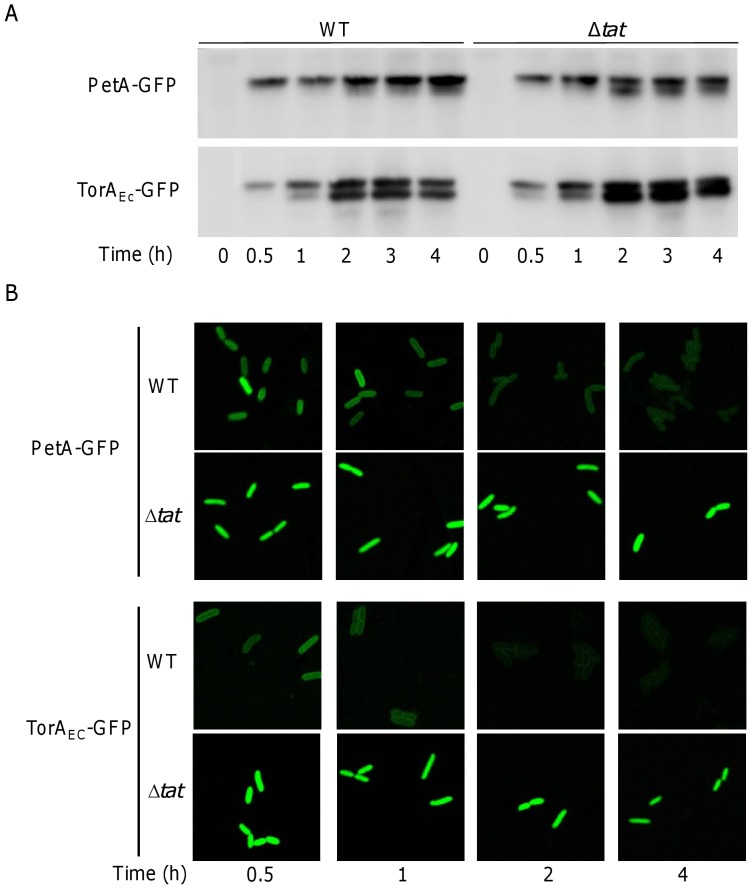
Cleavage of Tat signal peptide of PetA is independent of Tat-mediated translocation. Cells at exponential phase (∼0.4 of OD_600_) were induced by 0.1 mM IPTG for 2 hours, and then sampled for subsequently analysis. **A.** Time course Western blotting of GFP fusions expressed in the wild type and Δ*tat* strains. Same amount of whole cell protein was loaded in each lane. **B.** Fluorescent images for time course of expression and localization of PetA-GFP and TorA_Ec_-GFP in the wild type and *tat* mutants. Refer to Methods for details.

Cleavage of the signal peptide of the Tat substrates normally occurs in the periplasm after translocation across the membrane [3], [44]. During our investigation, we noticed that the fluorescence intensity of both TorA*_Ec_*-GFP and PetA-GFP in the *tat* ^+^ strain was significantly lower than that in the *tat* ^−^ strains (Fig. 3 and 5), implying that the GFP proteins free of signal peptides are not fluorescent in the periplasm, a scenario reported for the GFP fusion proteins transported by the Sec pathway [45], [46]. To confirm this, we examined the GFP fusions with antibodies against GFP in both *tat* ^+^ and *tat* ^−^ strains (Fig. 6A). Strikingly, two bands, corresponding to GFP proteins with and without the signal peptide respectively, were observed in all tested strains, suggesting that the fused GFP proteins were subjected to signal peptide cleavage regardless of their location. However, it is apparent that the removal of both TorA*_Ec_* and PetA signal peptides in the *tat* ^+^ strain were significantly slower than in the *tat* ^−^ strain. This can be readily explained by that certain time is needed for a portion of GFP fusion proteins to be transported to the periplasm before splitting into two proteins in the *tat* ^+^ strain. Furthermore, the cleavage of PetA signal peptides progressed by a rate significantly slower when compared to those of the TorA*_Ec_*, resulting in that the majority of PetA-GFP fusions remained intact.

If GFP proteins free of signal peptides are fluorescent in the cytoplasm but not in the periplasm, we would expect that the signal decay in the *tat* ^+^ strains should outpace that in the *tat* ^−^ strains. To test this, we monitored the fluorescence intensity of the wild type and *tat* mutant cells over time. As shown in Fig. 6B, the signal intensity by TorA*_Ec_*-GFP and PetA-GFP proteins in the Δ*tat* strain was constant during the testing period of 4 h, suggesting that GFPs in all forms are fluorescent as long as they are in the cytoplasm. In contrast, the signal intensity of both GFP fusions declined with time in the wild type strain. As GFP proteins exist in either fused form or GFP alone by Western blotting, these data, strongly suggest that GFP proteins free of signal peptides are fluorescent in the cytoplasm only. It is worth mentioning that TorA*_Ec_*-GFP showed an apparently more rapid decay rate for signals than PetA-GFP, implying that the PetA signal peptides are resistant to cleavage. This is not surprising because PetA has been reported to be inserted into the membrane with the uncleaved twin-arginine signal sequence as the transmembrane anchor [47], [48].

## Discussion

The investigation commenced with the identification of genes that have a significant impact on aerobic growth of *S. oneidensis* using GAF, a high-throughput approach enabling detection of mutations of interest in a library by means of a combination of transposon mutagenesis and microarray technology [24]. For all high-throughput technologies, it is important to reduce the number of false positives. Given that GAF copes with a mutant library rather than a single strain, the selection criteria adopted here are especially stringent. As a consequence, this T7-promoter-based GAF, which has the highest sensitivity and reproducibility [49], identified only 96 genes that met the selection criteria for attenuated or enriched replication. Conceivably, a considerable number of genes that have an impact on aerobic growth are missed out, such as *petA* and *petC*. Nevertheless, GAF revealed both *tatA* and *tatC*, indicating that the method is robust in identification of genes of interest provided experiments are well designed.

Owing to the fundamental role of the Tat export pathway in many cellular processes, particularly respiratory and photosynthetic energy metabolism and pathogenesis [1], [3], [5], [17], the system has been under intensive investigation for many years. This has led to important insights into the structure and function of the Tat complex and the mechanisms by which its substrates are recognized, translocated across the cytoplasmic membrane, and processed to the active form. Despite this, much still remains to be learned about the physiological function of the system because its substrates vary substantially in different organisms [3], [5], [17]. In the presented work, we have characterized the Tat machinery of *S. oneidensis* which carried unique features with respect to both structure and biochemical function. We present evidence to suggest that the Tat machinery is responsible for transport of PetA across the membrane, allowing cells to respire on oxygen by completing electron transfer to the terminal oxidases.

In addition to utilizing oxygen as a terminal electron acceptor (EA), *S. oneidensis* can anaerobically respire on various organic and inorganic substrates, including fumarate, nitrate, nitrite, DMSO, Fe(III), Mn(III) and (IV), Cr(VI), and U(VI), to name a few [18]. Correspondingly, the *S. oneidensis* genome encodes a large number of terminal reductases and associated proteins that contain cofactors such as Fe-S, molybdopterin, and NiFe, most of which are inserted enzymatically in the cytoplasm. It is therefore not surprising that some of these proteins are substrates of the Tat system. As *S. oneidensis* possesses a large number of metabolic pathways functioning in parallel, most of the predicted Tat substrates individually have undetectable or modest impact on the bacterial growth. However, loss of PetA impedes growth with oxygen significantly. The protein is one of essential subunits of the cytochrome *bc*_1_ complex, which transfers electrons from a low-potential quinol to a higher-potential *c*-type cytochrome [50]. The crucial role of the cytochrome *bc*_1_ complex in aerobic growth of *S. oneidensis* is further supported by the finding that strains deficient for PetC (another essential subunit of the cytochrome *bc*_1_ complex) and *cbb*_3_ cytochrome (the predominant terminal oxidase) displayed a similar level of growth defect under aerobic conditions [20]. Nevertheless, the *petA* mutant had a defect in aerobic growth less severe than the *tat* ^−^ strain, suggesting that growth defect of the *tat* ^−^ strain results from an additive effect of mislocalization of multiple substrates. These substrates may not be necessarily included in the 24 predicted proteins, as Tat substrates lacking the conserved RR dipeptide have been found [43], [51]. Moreover, the Tat pathway translocates a number of hetero-oligomeric protein complexes, of which only a single subunit carries an RR-signal sequence [52], [53]. Therefore, the repertoire of the natural Tat substrates in *S. oneidensis* should be larger.

The Tat pathway can assemble some membrane-bound proteins, such as C-tail anchored *E. coli* HybO, HyaA, HybA, FdnH, and FdoH, N-terminal signal anchored Rieske Fe-S proteins in both bacteria and plants [33]. The combined data of this study provide the clear evidence that the signal peptide in *S. oneidensis* PetA is resistant, at least to some extent, to cleavage after transport. This observation resonates with a couple of recent findings. In chloroplasts, *Paracoccus denitrificans*, and *Legionella pneumophila*, signal peptides of the Rieske Fe-S proteins are uncleavable because they play dual roles, as a Tat signal and as a membrane anchor [48], [54], [55]. An underlying mechanism proposed recently is that these signal peptides (in the case of *S. oneidensis* PetA, G-R-R-R-F-L-T) lack the consensus C-terminal lysine within the RR-signal peptide motif (S/T-R-R-X-F-L-K) or a ‘Sec-avoidance’ signal [48].

In the Tat complexes composed of TatA, TatB, and TatC characterized thus far, all three subunits are essential for the transport of endogenous Tat substrates [3]. TatA and TatB, apparently resulted from gene duplication in evolution, are structural homologues. Consequently, single amino acid changes enable *E. coli* TatA to simultaneously substitute for both TatA and TatB function, and moreover, TatA from *Bacillus subtilis*, whose complex consists of TatA and TatC only, is able to take place of either TatA or TatB in *E. coli* [6], [56]. As the essential subunits of the *S. oneidensis* Tat machinery include TatA and TatC but not TatB, this system appears to represent a new twist on the assembly of the complex. The *S. oneidensis* TatB (149 a.a.) and its *E. coli* counterpart (162 a.a.) are N-terminal membrane anchored and display a high level of similarity in structure. Although loss of TatB did not show any impact on the relevant physiological aspects through our characterization, it is not possible to rule out that TatB plays a subtle/specific role for transport of certain substrates. To determine whether dispensability of TatB in a TatABC system is a widespread phenomenon definitely and intriguingly merits.

## Methods

### Bacterial strains, plasmids, and growth conditions

A list of all bacterial strains and plasmids used in this study is given in Table 2. For genetic manipulation, *E. coli* and *S. oneidensis* strains were grown in Luria-Bertani (LB) medium under aerobic conditions at 37 or 30°C, respectively. When needed, the antibiotics were supplemented at the following concentrations: ampicillin, 50 µg/mL; kanamycin, 50 µg/mL, and gentamycin, at 15 µg/mL. Primers used for generating PCR products in this study are given in Table S1.

**Table 2:** Bacterial strains and plasmids used in this study.

Strain or plasmid	Description	Reference or source
*E. coli* strains		
DH5α	Host for regular cloning	Lab stock
WM3064	Donor strain for conjugation; Δ*dapA*	W. Metcalf, UIUC
*S. oneidensis* strains		
MR-1	Wild type	Lab stock
HG4202	*tatA* in-frame mutant derived from MR-1; Δ*tatA*	This study
HG4203	*tatB* in-frame mutant derived from MR-1; Δ*tatB*	This study
HG4204	*tatC* in-frame mutant derived from MR-1; Δ*tatC*	This study
HG4206	*tatD* in-frame mutant derived from MR-1; Δ*tatD*	This study
HGTatABC	*tatABC* in-frame mutant derived from MR-1; Δ*tat* (Δ*tatABC*)	This study
HG0101	*fdnG* in-frame mutant derived from MR-1; Δ*fdnG*	This study
HG0483	*sirC* in-frame mutant derived from MR-1; Δ*sirC*	This study
HG0608	*petA* in-frame mutant derived from MR-1; Δ*petA*	This study
HG0847	*napG* in-frame mutant derived from MR-1; Δ*napG*	This study
HG0848	*napA* in-frame mutant derived from MR-1; Δ*napA*	This study
HG0881	*SO0881* in-frame mutant derived from MR-1; Δ*SO0881*	This study
HG0939	*SO0939* in-frame mutant derived from MR-1; Δ*SO0939*	This study
HG1232	*torA* in-frame mutant derived from MR-1; Δ*torA*	This study
HG1405	*SO1405* in-frame mutant derived from MR-1; Δ*SO1405*	This study
HG1414	*SO1414* in-frame mutant derived from MR-1; Δ*SO1414*	This study
HG1429	*dmsA* in-frame mutant derived from MR-1; Δ*dmsA*	This study
HG2385	*phoX* in-frame mutant derived from MR-1; Δ*phoX*	This study
HG3048	*iorB* in-frame mutant derived from MR-1; Δ*iorB*	This study
HG3058	*SO3058* in-frame mutant derived from MR-1; Δ*SO3058*	This study
HG3301	*SO3301* in-frame mutant derived from MR-1; Δ*SO3301*	This study
HG3791	*SO3791* in-frame mutant derived from MR-1; Δ*SO3791*	This study
HG3921	*hydB* in-frame mutant derived from MR-1; Δ*hydB*	This study
HG4062	*phsA* in-frame mutant derived from MR-1; Δ*phsA*	This study
HG4151	*SO4151* in-frame mutant derived from MR-1; Δ*SO4151*	This study
HG4358	*dmaA-2* in-frame mutant derived from MR-1; Δ*dmaA-2*	This study
HG4508	*SO4508* in-frame mutant derived from MR-1; Δ*SO4508*	This study
HG4509	*SO4509* in-frame mutant derived from MR-1; Δ*SO4509*	This study
HG4512	*SO4512* in-frame mutant derived from MR-1; Δ*SO4512*	This study
HG4513	*SO4513* in-frame mutant derived from MR-1; Δ*SO4513*	This study
HGform	*SO4509-4513* in-frame mutant derived from ΔSO*4509*; Δ*form*	This study
HGflav	*SO*3058-1414-3301 in-frame mutant derived from ΔSO*3058-1414*; Δ*flav*	This study
HGform-608	*SO4509-4513* in-frame mutant derived from ΔSO*4509*; Δ*petA*Δ*form*	This study
HGflav-608	*SO*3058-1414-3301 in-frame mutant derived from ΔSO*3058-1414*; Δ*petA*Δ*flav*	This study
Plasmids		
pBSL180	Km^r^, Mobilizable suicide vector; modified Tn*10*	[25]
pHGT214	pBSL180 derivative, GAF vector containing T_7_ promoter linked to restriction sequences	This study
pDS3.0	Ap^r^, Gm^r^, derivative from suicide vector pCVD442	Lab stock
pHG101	Km^r^, promoterless broad host vector used for complementation	[35]
pHG102	pHG101 containing the *arcA* promoter	[35]
pHG101-Tat	pHG101 containing the *tatABC* operon	This study
pHG101-TatA	pHG101 containing *tatA*	This study
pHG102-TatC	pHG102 containing *tatC*	This study
pHG101-PetA	pHG101 containing *petA*	This study
pHG101-PetA^KK^	PetA signal peptide consensus region R-R replaced by K-K; derived from pHG101-PetA	This study
pBBR1MCS-2	Km^r^,	[58]
pBADHisA	Ap^r^, the *rrnB* transcription termination sequence donor vector	Lab stock
pET-28a	Ap^r^, the *lacI* donor vector	Lab stock
pHGE-P*tac*	Km^r^, broad host expression vector derived from pBBR1MCS2	This study
pHGE-P*tac*-PetAGFP	Km^r^, pHGE-Ptac containg PetAsp-GFP	This study
pHGE-P*tac*TorAGFP	Km^r^, pHGE-Ptac containg TorAsp-GFP	This study

### Physiological characterization

M1 defined medium containing 0.02% (w/v) of vitamin-free Casamino Acids and 30 mM lactate (pH 7.4) was used unless otherwise noted [29]. Fresh medium was inoculated with overnight cultures grown from a single colony by 1∶100 dilution, incubated on a shaker at 250 rpm at 30°C under aerobic conditions, and growth was measured by recording cell densities of cultures at 600 nm. For anaerobic growth, *S. oneidensis* strains were grown on M1 medium supplemented one of the following electron acceptors: NaNO_2_, 3 mM and DMSO, 50 mM.

Morphology of cells was examined with a Motic BA310 phase-contrast microscope. Micrographs were captured with a Moticam 2306 charged-coupled-device camera and Motic images advanced 3.2 software. All experiments were conducted at least in triplicate. Motility assay on swimming LB plates (0.25% agar) and pellicle formation assay in LB broth were performed essential as described previously [34], [35]. In brief, mid-log-phase cultures were adjusted to an equivalent OD_600_ of 0.4 for each strain with fresh LB broth, five-microliter of which was spotted onto swimming plates by piercing it with a thin pipette tip. Plates were incubated at 30°C. Cultures at exponential phase (∼0.6 of OD_600_) were diluted 1∶100 with fresh LB broth, aliquotted into 24-well plates (2 ml/well), and incubated at 30°C to test the pellicle formation. For sodium dodecyl sulfate (SDS) sensitivity assay, strains were grown aerobically in LB broth to an OD_600_ of 0.6. Cultures at exponential phase (∼0.6 of OD_600_) were adjusted to 10^4^, 10^5^, 10^6^, 10^7^ cells per milliliter, and 10 µl of each dilution was dripped on the plates contained SDS of various concentrations. Plates were incubated 30°C and photos were taken in a time course manner.

### Genomic array footprinting analysis for *S. oneidensis*

For Genomic array footprinting (GAF) analysis, a plasmid, pHGT214, was constructed using the backbone of pBSL180, whose characteristics make it the ideal vector to perform transposon mutagenesis in *S. oneidensis* [25], [57]. Plasmid pHGT214 is virtually pBSL180 with addition of a fragment containing T7 promoter next to a GGATCC site generated by annealing two oligonucleotides (T7-F/R). The restriction site can be recognized by a variety of enzymes, including *Bam*HI, *Bst*YI, *Dpn*I, *Dpn*II, and *Sau*3AI. Given that the ideal DNA fragments for subsequent steps of genetic footprinting are blunt-ended and 400 bp in length [23], *Dpn*I was chosen for the study. The *Dpn*I-digested genomic DNAs were used as the template for *in vitro* transcription for the subsequent experiments.

Plasmid pHGT214 was transferred from *E. coli* WM3064 to *S. oneidensis* by conjugation, resulting in a random library of transposon-generated mutants. A population of approximately 10^5^ independent mutants was pooled, aliquoted, and saved, and used as the control sample (S_0_) when necessary. Three aliquots as biological replicates were subjected to competition experiments under aerobic conditions according to the method described previously [26]. In brief, each aliquot was inoculated into 100 ml media and incubated at 200 rpm 30°C. After 24 h, 1 ml of the competing cells was transferred into fresh 99 ml of the same medium and the rest was taken as the sample of S_1_. Repeat the experiment the next day and collect the sample as S_2_. In total, the procedure was repeated for 5 consecutive days. The genomic DNAs were extracted, quantified, and digested with *Dpn*I. After purification, 1 µg of the digested DNA was used as the template for *in vitro* transcription with the MEGAscript T7 transcription kit (Ambion) in a total volume of 50 µl, which was subsequently dye-labeled by reverse transcription as described elsewhere [27]. These labeled cDNAs were co-hybridized with those generated from the control sample S_0_ onto the *S. oneidensis* whole genome microarrays and data were analyzed as previously described [27]–[29]. The microarray data were deposited in the NCBI Gene Expression Omnibus (GEO) web-based data repository (ID: GSE42479).

### Construction and complementation of in-frame deletion mutants

In this study, in-frame deletion strains were constructed using the Fusion PCR method and primer information is available upon request. In brief, two fragments flanking the targeted gene were amplified independently first and joined together by the second round of PCR. The resulting fusion fragment for each individual gene was introduced into plasmid pDS3.0. The mutagenesis vector was transformed into *E. coli* WM3064, which then conjugated into *S. oneidensis*. Integration of the mutagenesis construct into chromosome was selected by gentamycin resistance and by PCR verification. Homologous recombination mutagenesis was triggered by growth in LB broth in the absence of NaCl. The gentamycin-sensitive and sucrose-resistant colonies were screened by PCR for deletion of the targeted gene.

For complementation of genes next to their promoter, a fragment containing the targeted gene and its native promoter was generated by PCR and cloned into pHG101 [35]. For other genes, the targeted gene was amplified and inserted into MCS of pHG102 under the control of the *arcA* promoter. Introduction of each verified complementation vector into the corresponding mutant was done by mating with WM3064 containing the vector, and confirmed by plasmid extraction and restriction enzyme mapping.

### Site-directed mutagenesis

The complementation plasmid pHG101-PetA was used as the template for site-directed mutagenesis. In brief, primers (PetA^KK^-F/R) were designed in which the PetA signal peptide consensus region R-R was changed to K-K. PCR products digested by *Dpn*I were transformed to *E. coli* WM3064. After sequencing verification, the resulting plasmid was conjugated into the Δ*petA* strain for characterization.

### Development of an IPTG-inducible expression system for *S. oneidensis*

An inducible expression system for *S. oneidensis* was developed in this study. Plasmid pBBR1MCS-2 [58] was digested with *Bst*BI and religated, resulting in pHG100. A DNA fragment containing P*tac* was generated by annealing oligonucleotides P*tac*-F/R and cloned into *age*I site of pHG100, producing pHGE-01. The *rrnB* transcription termination sequence was PCR amplified with primers (Term-F/R) using pBADHisA (Invitrogen) as template and cloned into *Eco*RI and *Age*I sites of pHGE-01, generating pHGE-02. *E. coli lacI* was then amplified with pET-28a as template and primers (ELacI-F/R) and inserted into the *Age*I site of pHGE-02, resulting in pHGE-P*tac*.

### Construction and expression of GFP fusions

To construct GFP fusion vectors, gene fragments of interest were PCR amplified with primers (PetA-F/R, TorA_Ec_-F/R, eGFP-F/R), which were designed such that the resultant products of PetA signal peptide and eGFP or TorA_Ec_ signal peptide and eGFP can be joined together by the second round of PCR. The PCR fusions were cloned into pHGE-P*tac* using standard methods, and transformed into *E. coli* WM3064. After verification by sequencing, the expression vectors were moved into *S. oneidensis* strains by conjugation. For expression of cloned GFP fusions, 0.1 mM isopropyl β-D-1-thiogalactopyranoside (IPTG) was added to mid-log phase cultures (∼0.4 of OD_600_). The cultures were incubated at 200 rpm at 30°C for 2 h.

### Microscopy

After induction, 100 µl of the culture was dropped onto a layer of 3% agar on a slide for immobilization. After the droplet dried, a glass coverslip was placed on top. Expression and localization of GFP fusions were visualized using a Zeiss LSM-510 confocal laser scanning microscope equipped with a 63× oil immersion objective (numerical aperture 1.4). GFP was excited using 488-nm irradiation from an argon ion laser and fluorescent emission was monitored by collection across windows of 505 to 530 nm.

### Protein analysis

SDS-polyacrylamide gel electrophoresis (SDS-PAGE) and Western blotting assay were performed as described previously [35]. Protein concentration was monitored by GE Nanovue Spectrophotometer and/or using a Bradford assay with BSA as a standard (Bio-Rad). In brief, mid-log phase cultures induced by 0.1 mM IPTG for 2 h were collected by centrifugation and the resulting cell pellets were washed twice with phosphate-buffered saline (PBS) and then subjected to SDS-PAGE (12%). After membrane transfer for 2 h at 60 V using a Criterion blotter (Bio-Rad), the blotting membrane was probed with the primary antibody Mouse Anti-eGFP-tag Monoclonal Antibody (GenScript) and then the second antibody Goat anti-Mouse IgG-HRP (Horse Radish Peroxidase) (Roche Diagnostics). Detection was performed using a chemiluminescence Western blotting kit (Roche Diagnostics) in accordance with the manufacturer's instructions and images were visualized with the UVP Imaging System.

### Bioinformatics and statistical analyses

Tat substrates in *S. oneidensis* were predicted using programs TatFind [36], PRED-TAT [37] and TatP [38]. Transmembrane protein topology was predicted using TMHMM [41]. Signal peptide motif sequences of Tat substrates for alignments were obtained from Genbank. Alignments were performed using Clustal Omiga (http://www.ebi.ac.uk/Tools/msa/clustalo). Sequence logos were generated using WebLogo [59]. Values are presented as means ± SD (standard deviation). Student's *t*-test was performed with statistical significance set at the 0.05 confidence level.

## Supporting Information

Figure S1 **Comparative analysis of phenotypes of** ***S. oneidensis tat*** **mutants.** **A.** Morphology by phase-contrast microscope. **B.** Motility phenotype of *tat* mutants. The plates were incubated at 30°C for 36 h to test the motility of different strains. Verified non-motile mutant strain Δ*fliD* was used as the negative control. **C.** Pellicle formation of *tat* mutants. The plates were incubated at 30°C for 24 h. Verified pellicle-free mutant strain Δ*aggA* was used as the negative control. **D.** Susceptibility of *tat* mutants to SDS. 0.25% SDS was used to test the sensitivity of *tat* mutants. For B, C, and D, photos were taken in a time course manner.
(PDF)

Table S1 **Primers used in this study.**
(PDF)

Table S2 **Genes whose abundance is significantly altered in GAF.**
(PDF)

Table S3 **Potential Tat substrates in** ***S. oneidensis*** **.**
(PDF)
