# Supplementary material for: Mislocalization of Rieske Protein PetA Predominantly Accounts for the Aerobic Growth Defect of tat Mutants in Shewanella oneidensis
Source: PLoS One. 2013 Apr 11;8(4):e62064. doi: 10.1371/journal.pone.0062064 (PMC3623810; doi:10.1371/journal.pone.0062064)
Supplement: Figure S1 — Comparative analysis of phenotypes of S. oneidensis tat mutants. A. Morphology by phase-contrast microscope. B. Motility phenotype of tat mutants. The plates were incubated at 30°C for 36 h to test the motility of different strains. Verified non-motile mutant strain ΔfliD was used as the negative control. C. Pellicle formation of tat mutants. The plates were incubated at 30°C for 24 h. Verified pellicle-free mutant strain ΔaggA was used as the negative control. D. Susceptibility of tat mutants to SDS. 0.25% SDS was used to test the sensitivity of tat mutants. For B, C, and D, photos were taken in a time course manner. (PDF) [file pone.0062064.s001.pdf]

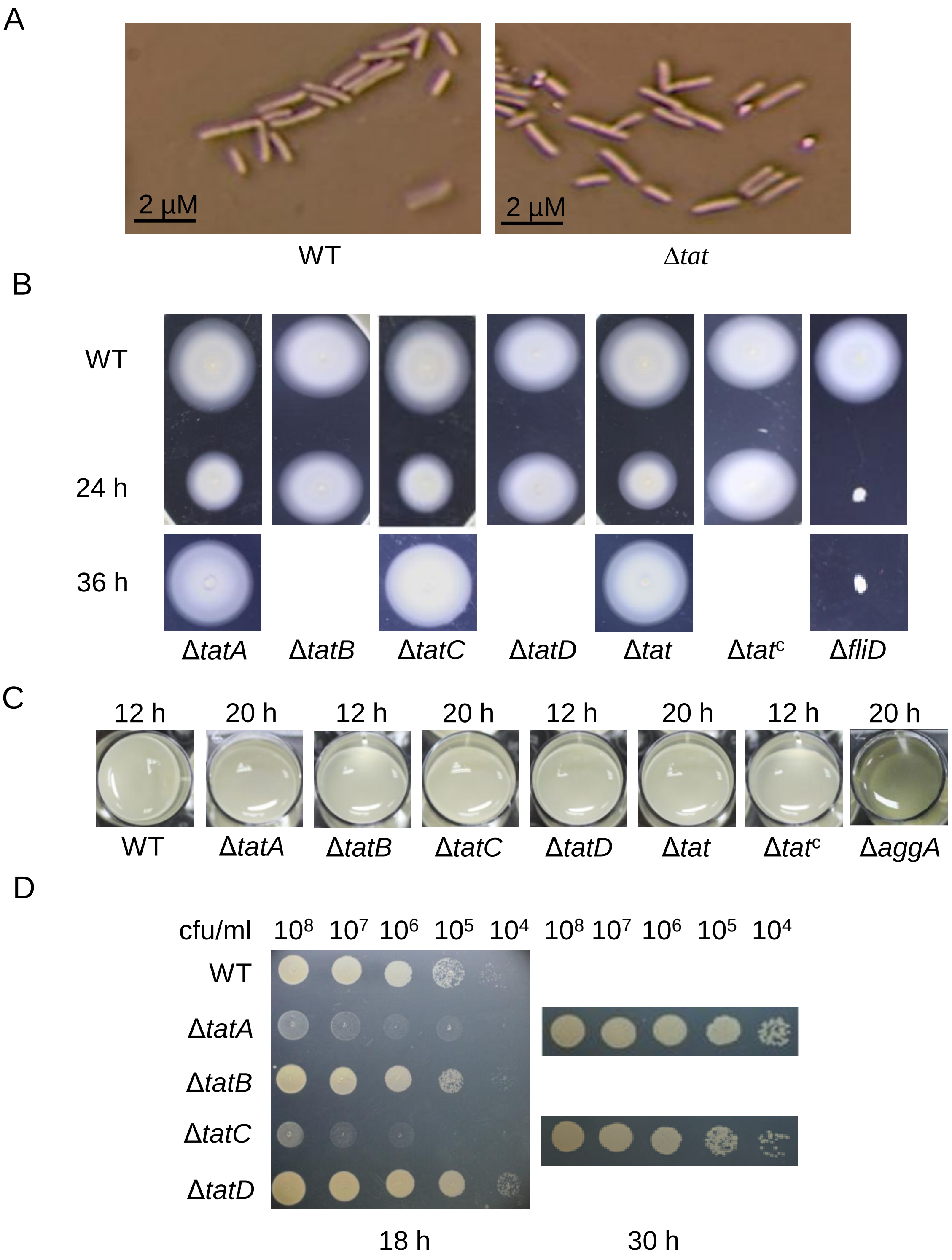

FIG. S1. Comparative analysis of phenotypes of *S. oneidensis tat* mutants. (A) Morphology by phase-contrast microscope. (B) Motility phenotype of *tat* mutants. The plates were incubated at 30°C for 36 h to test the motility of different strains. Verified non-motile mutant strain Δ*fliD* was used as the negative control. (C) Pellicle formation of *tat* mutants. The plates were incubated at 30°C for 24 h. Verified pellicle-free mutant strain Δ*aggA* was used as the negative control. (D) Susceptibility of *tat* mutants to SDS. 0.25% SDS was used to test the sensitivity of *tat* mutants. For (B), (C) and (D), photos were taken in a time course manner.
