# Supplementary material for: Mislocalization of Rieske Protein PetA Predominantly Accounts for the Aerobic Growth Defect of tat Mutants in Shewanella oneidensis
Source: PLoS One. 2013 Apr 11;8(4):e62064. doi: 10.1371/journal.pone.0062064 (PMC3623810; doi:10.1371/journal.pone.0062064)
Supplement: Table S1 — Primers used in this study. (PDF) [file pone.0062064.s002.pdf]

TABLE S1. Primers used in this study

**GAF**	
T7-F	CGGATCCCACCTAAGTGAATTGTAATACGACTCACTATAGGGCGAT
T7-R	CATGGCCTAGGGTGGATTCACTTAACATTATGCTGAGTGATATCCCGCTAGATC
**Complementation**	
SO0608-CF	GGAATTCCGAAATCGCTCAGAATGGGTTCCT
SO0608-CR	CGGGATCCACGTCGCCGTCATTGGGATGC
TatABC-CF	GGAATTCATTGCTGAGTTCTCAGCAACAA
TatABC-CR	GGAATTCTATGGCGGCAGTCAGTAGAGTA
TatA-CF	GGAATTCATTGCTGAGTTCTCAGCAACAA
TatA-CR	CGGGATCCCAAACATAAAGAGTTACGCCTG
TatC-CF	GGAATTCATGCGTTTTACTCTACTGACTGC
TatC-CR	GGAATTCTTATTTGAGGCGCTTTACCCTC
**Site-directed mutagenesis**	
PetA^{KK}-F	GTCGATACCGGAAAGAAGAGATTCCTGACAGCCGCAACCGCC
PetA^{KK}-R	TGGCGCATTGCTCATCCACTTATCTCCCAGAGGGTGTTGG
**Plasmid construction**	
Ptac-F	CCGGAGCTGTTGACAATTAATCATCGGCTCGTATAATGTGTGGAATTGTGAGCGGATAACAATTTC ACACAGGAGAGAATTCATG
Ptac-R	CCGGTCATGAATTCTCTCCTGTGTGAAATTGTTATCCGCTCACAATTCCACACATTATACGAGCCG ATGATTAATTGTCAACAGCT
Term-F	TAAGAATTCATGGAGCTCGAGGATCCAAGCTTGGCTGTTTTGGCGGATG
Term-R	GGTACCGGTGCGTTCACCGACAAACA
ELacI-F	AAACCGGTGACACCATCGAATGGCGC
ELacI-R	AAACCGGTGCCTAATGAGTGAGCT

**Protein expression**	
PetAsp-F	GGAATTCATGAGCAATGCGCCAGTCGATACC
PetAsp-R	AAAGTTCTTCTCCTTTGCTAGCCATACCTGCAGCTTTCGCCTTGGCACTC
TorAsp-F	GGAATTCATGAACAATAACGATCTCTTTC
TorAsp-R	AAAGTTCTTCTCCTTTGCTAGCCATCGCCGCTTGCGCCGCAGT
eGFP-F	ATGGCTAGCAAAGGAGAAGAACTTTTC
eGFP-R	GGAATTCTTATTTGTAGAGCTCATCCATGCC
