# Supplementary material for: Mislocalization of Rieske Protein PetA Predominantly Accounts for the Aerobic Growth Defect of tat Mutants in Shewanella oneidensis
Source: PLoS One. 2013 Apr 11;8(4):e62064. doi: 10.1371/journal.pone.0062064 (PMC3623810; doi:10.1371/journal.pone.0062064)
Supplement: Table S2 — Genes whose abundance is significantly altered in GAF. (PDF) [file pone.0062064.s003.pdf]

TABLE S2. Genes whose abundance is significantly altered in GAF

Locus	Gene	S05/S01	*p* value	Predicted function
SO0013	*SO0013*	0.418	0.014	hypothetical protein
SO0209	*SO0209*	0.413	0.013	hypothetical protein
SO0275	*argC*	0.498	0.031	N-acetyl-gamma-glutamyl-phosphate reductase
SO0293	*gph*	0.306	0.000	phosphoglycolate phosphatase
SO0294	*trpS*	0.482	0.028	tryptophanyl-tRNA synthetase
SO0313	*potE*	0.402	0.011	putrescine-ornithine antiporter
SO0344	*prpC*	3.197	0.043	methylcitrate synthase
SO0358	*SO0358*	0.472	0.025	endoribonuclease L-PSP, putative
SO0403	*SO0403*	0.285	0.000	hypothetical protein
SO0442	*purH*	0.379	0.000	phosphoribosylaminoimidazolecarboxamide formyltransferase/IMP cyclohydrolase
SO0468	*ubiA*	0.434	0.016	4-hydroxybenzoate polyprenyl transferase
SO0526	*SO0526*	0.491	0.030	acetyltransferase, GNAT family
SO0541	*SO0541*	0.467	0.025	metallo-beta-lactamase family protein
SO0632	*hrpB*	0.390	0.000	ATP-dependent helicase HrpB
SO0683	*SO0683*	0.381	0.000	hypothetical protein
SO0718	*SO0718*	0.447	0.019	conserved hypothetical protein
SO0778	*SO0778*	0.403	0.011	oxidoreductase, FAD-binding, UbiH/Coq6 family
SO0816	*SO0816*	0.456	0.020	hypothetical protein
SO0920	*SO0920*	0.486	0.030	acetyltransferase, GNAT family
SO0941	*SO0941*	0.340	0.000	hypothetical protein
SO1063	*slyX*	0.444	0.018	SlyX protein
SO1150	*rpiA*	0.409	0.012	ribose 5-phosphate isomerase
SO1177	*cutE*	2.235	0.034	apolipoprotein N-acyltransferase
SO1265	*SO1265*	0.303	0.000	transcriptional regulator, putative
SO1351	*pdxJ*	0.418	0.014	pyridoxal phosphate biosynthetic protein PdxJ
SO1352	*acpS*	0.391	0.000	holo-(acyl-carrier protein) synthase
SO1373	*SO1373*	0.426	0.015	conserved hypothetical protein
SO1378	*SO1378*	2.029	0.032	ThiJ/PfpI family protein
SO1416	*SO1416*	0.367	0.000	DNA-binding response regulator
SO1519	*SO1519*	0.361	0.000	iron-sulfur cluster-binding protein
SO1530	*pomB*	0.430	0.015	sodium-driven polar flagellar protein PomB
SO1556	*SO1556*	0.498	0.031	conserved hypothetical protein
SO1640	*fabZ*	0.461	0.022	(3R)-hydroxymyristoyl-(acyl-carrier-protein) dehydratase

SO1728	*SO1728*	0.408	0.012	hypothetical protein
SO1763	*sat*	2.462	0.039	streptogramin A acetyl transferase
SO1865	*SO1865*	0.411	0.012	ABC transporter, ATP-binding protein
SO1911	*SO1911*	0.391	0.000	oxidoreductase, short chain dehydrogenase/reductase family
SO1928	*sdhA*	0.183	0.006	succinate dehydrogenase, flavoprotein subunit
SO1929	*sdhB*	0.362	0.043	succinate dehydrogenase, iron-sulfur protein
SO1932	*sucC*	0.387	0.040	succinyl-CoA synthase, beta subunit
SO1945	*phoQ*	0.357	0.000	sensor protein PhoQ
SO2059	*SO2059*	0.148	0.000	hypothetical protein
SO2098	*hyaB*	0.067	0.000	quinone-reactive Ni/Fe hydrogenase, large subunit
SO2188	*SO2188*	2.085	0.033	polyphosphate kinase, truncation
SO2260	*suhB*	2.654	0.041	extragenic suppressor protein SuhB
SO2339	*SO2339*	2.484	0.040	alpha keto acid dehydrogenase complex, E1 component, alpha subunit
SO2357	*SO2357*	0.477	0.027	conserved hypothetical protein
SO2361	*ccoP*	0.418	0.037	cytochrome c oxidase, cbb3-type, subunit III
SO2427	*SO2427*	3.373	0.045	TonB-dependent receptor, putative
SO2532	*SO2532*	0.494	0.031	methylated-DNA--protein-cysteine methyltransferase, putative
SO2542	*SO2542*	0.483	0.029	conserved hypothetical protein
SO2588	*SO2588*	2.062	0.032	protein-methionine-S-oxide reductase, PilB family
SO2650	*SO2650*	0.465	0.024	conserved hypothetical protein
SO2706	*astB*	3.374	0.044	succinylarginine dihydrolase
SO2752	*SO2752*	0.477	0.028	conserved hypothetical protein TIGR00051
SO2796	*SO2796*	0.435	0.016	conserved hypothetical protein
SO2842	*SO2842*	0.440	0.017	peptidase, M23/M37 family
SO2907	*SO2907*	7.325	0.006	TonB-dependent receptor domain protein
SO2923	*gltS*	0.394	0.000	sodium/glutamate symporter
SO3096	*SO3096*	0.460	0.022	RNA polymerase sigma-70 factor, ECF subfamily
SO3105	*pspE-1*	0.367	0.000	phage shock protein E
SO3156	*SO3156*	0.373	0.000	hypothetical protein
SO3217	*mdh*	0.340	0.000	flagellar biosynthetic protein FliQ
SO3332	*SO3332*	0.457	0.021	transcriptional regulator, CopG family
SO3361	*SO3361*	2.336	0.034	conserved hypothetical protein
SO3393	*SO3393*	0.343	0.000	transcriptional regulator, TetR family
SO3441	*pyrG*	0.388	0.000	CTP synthase
SO3534	*mviN*	0.402	0.010	MviN protein

SO3553	*SO3553*	0.407	0.011	sulfate permease family protein
SO3667	*SO3667*	5.134	0.039	conserved hypothetical protein
SO3695	*pyrC*	0.416	0.013	dihydroorotase, homodimeric type
SO3698	*SO3698*	0.476	0.027	hypothetical protein
SO3800	*aceE*	0.301	0.000	serine protease, subtilase family
SO3822	*SO3822*	0.455	0.019	conserved hypothetical protein
SO3898	*SO3898*	0.313	0.000	L-sorbosone dehydrogenase, putative
SO3913	*SO3913*	2.224	0.033	conserved hypothetical protein
SO3922	*SO3922*	0.445	0.018	formate dehydrogenase, putative
SO4029	*SO4029*	0.142	0.000	transporter, putative
SO4043	*SO4043*	2.624	0.040	TonB domain protein
SO4044	*SO4044*	0.484	0.029	hypothetical protein
SO4048	*SO4048*	0.288	0.000	cytochrome c family protein
SO4050	*SO4050*	0.347	0.000	conserved hypothetical protein
SO4072	*yliG*	2.408	0.038	MiaB-like putative RNA modifying enzyme YliG
SO4113	*mshK*	0.415	0.013	MSHA biogenesis protein MshK
SO4202	*tatA*	0.370	0.000	TatA
SO4204	*tatC*	0.395	0.000	TatC
SO4252	*SO4252*	3.246	0.042	prolyl oligopeptidase family protein
SO4263	*SO4263*	0.396	0.010	conserved hypothetical protein
SO4329	*SO4329*	0.472	0.026	conserved hypothetical protein
SO4480	*aldA*	2.387	0.037	aldehyde dehydrogenase
SO4511	*SO4511*	0.266	0.000	formate dehydrogenase, C subunit, putative
SO4562	*SO4562*	0.328	0.000	conserved hypothetical protein
SO4578	*SO4578*	0.438	0.017	transposase, putative
SO4617	*dinF*	0.356	0.000	DNA-damage-inducible protein F
SO4729	*SO4729*	0.462	0.023	conserved hypothetical protein
SOA0123	*SOA0123*	0.484	0.029	conserved domain protein
