# Supplementary material for: Mislocalization of Rieske Protein PetA Predominantly Accounts for the Aerobic Growth Defect of tat Mutants in Shewanella oneidensis
Source: PLoS One. 2013 Apr 11;8(4):e62064. doi: 10.1371/journal.pone.0062064 (PMC3623810; doi:10.1371/journal.pone.0062064)
Supplement: Table S3 — Potential Tat substrates in S. oneidensis . (PDF) [file pone.0062064.s004.pdf]

TABLE S3. Potential Tat substrates in *S. oneidensis*

Locus	Gene name	Product	Prediction Software[a]		
			TatFind 1.4	PRED-TAT	TatP 1.0[b]
SO0101	*fdnG*	nitrate-inducible formate dehydrogenase, molybdopterin-binding subunit, FdnG (programmed recoding with selenocysteine,insertion of selenocysteine at internal stop codon;)	Y	Y	5
SO0102	*fdnH*	nitrate-inducible formate dehydrogenase, iron-sulfur subunit, FdnH			4
SO0403		expressed outer membrane protein		Y	
SO0433	*rsd*	regulator of sigma D, Rsd	Y	Y	
SO0483	*sirC*	thiosulfate-inducible NrfC-like 4Fe-4S ferredoxin	Y	Y	
SO0519	*czcB_1*	subunit, CzcB_1			4
SO0663		prophage MuSo1 conserved hypothetical protein	Y	Y	
SO0608	*petA*	PetA	Y	Y	4
SO0715	*sorA*	sulfite dehydrogenase, molybdopterin subunit, SorA		Y	
SO0729		hypothetical protein		Y	
SO0764		expressed periplasmic protein			5
SO0806		alkaline phosphatase family protein		Y	
SO0809	*azu*	periplasmic azurin, Azu			4
SO0833	*endA*	periplasmic endonuclease I, EndA			4
SO0847	*napG*	periplasmic nitrate reductase, ferredoxin component, NapG	Y	Y	4
SO0848	*napA*	periplasmic nitrate reductase, molybdopterin-binding subunit, NapA	Y	Y	5
SO0881		expressed periplasmic protein	Y	Y	
SO0939		split-soret diheme cytochrome c	Y	Y	5
SO0970	*fccA*	periplasmic fumarate reductase, FccA			4
SO0986		chromate transporter, CHR family			4
SO1006		dienelactone hydrolase family protein		Y	
SO1168	*mrdA*	penicillin-binding protein 2, MrdA		Y	
SO1199	*glmM*	phosphoglucosamine mutase, GlmM			4
SO1232	*torA*	trimethylamine-N-oxide reductase, TorA	Y	Y	5
SO1276	*puuE*	GABA aminotransferase, PLP-dependent, PuuE		Y	5
SO1405		transglutaminase family protein	Y	Y	4
SO1414		flavocytochrome c flavin subunit, putative	Y	Y	5
SO1429	*dmaA-1*	outer membrane dimethyl sulfoxide reductase, molybdopterin-binding subunit, DmsA	Y	Y	
SO1677	*acaT*	3-ketoacyl-CoA thiolase, AcaT			4
SO2042	*yedY*	periplasmic oxidoreductase, molybdenum-binding subunit, YedY		Y	
SO2065		tyrosine transporter, TyrP			5
SO2099	*hyaA*	NiFe hydrogenase, small subunit, HyaA		Y	
SO2110		secreted protein of unknown function DUF882		Y	
SO2232		hypothetical protein			4
SO2385	*phoX*	monomeric alkaline phosphatase, PhoX	Y	Y	
SO2467		adhesion-related protein			4
SO2523		TonB-dependent receptor			4
SO2525		ABC transporter, ATP-binding protein			4
SO2577	*minD*	septum site-determining protein, MinD			4
SO2679	*E_2*	prophage MuSo2 large terminase subunit, E_2		Y	

SO2715		TonB-dependent receptor			5
SO2753		prolyl endopeptidase			4
SO2804		hypothetical inner membrane protein		Y	
SO2813		oxidoreductase, short chain dehydrogenase/reductase family			4
SO2876		conserved hypothetical lipoprotein		Y	
SO2935		oxidoreductase, short-chain dehydrogenase/reductase family			4
SO3006		prophage LambdaSo C-5 cytosine-specific DNA methylase			5
SO3048	*iorB*	isoquinoline 1-oxidoreductase, molybdopterin-binding subunit, IorB	Y	Y	5
SO3058		flavocytochrome c, flavin subunit	Y	Y	4
SO3109		expressed protein with zinc-finger-like domain			4
SO3120		binding)		Y	
SO3156.1		low complexity protein of unknown function			5
SO3193	*otnA*	OtnA			4
SO3301		flavocytochrome c, flavin subunit	Y	Y	
SO3537	*rpsT*	ribosomal protein S20, RpsT		Y	
SO3791		peptidase, M19 family	Y	Y	5
SO3909		conserved hypothetical inner membrane protein			4
SO3914		TonB-dependent siderophore receptor			4
SO3921	*hydB*	HydB	Y	Y	4
SO3939	*rpsI*	ribosomal protein S9, RpsI			4
SO4062	*phsA(psrA)*	sulfur reductase, molybdoperterin-binding subunit, PhsA	Y	Y	4
SO4151		secreted polysaccharide deacetylase	Y	Y	4
SO4219	*murG*	undecaprenyldiphospho-muramoylpentapeptide beta-N-acetylglucosaminyltransferase, MurG			5
SO4339		sodium-dependent transporter, NSS family			5
SO4358	*dmaA-2*	outer membrane oxidoreductase, molybdopterin-binding subunit	Y	Y	
SO4383	*fabF_2*	3-oxoacyl-(acyl-carrier-protein) synthase II, FabF_2		Y	
SO4405	*katG_2*	catalase/peroxidase HPI, KatG_2			4
SO4508		formate dehydrogenase accessory protein	Y	Y	5
SO4509		formate dehydrogenase, molybdopterin-binding subunit, FdhA_1	Y	Y	
SO4512		formate dehydrogenase accessory protein	Y	Y	
SO4513		formate dehydrogenase, molybdopterin-binding subunit, FdhA_2	Y	Y	5
SO4748	*atpG*	ATP synthase F1, gamma subunit, AtpG			4

[a] These three prediction softwares were used to screen the genome sequence of S. oneidensis MR-1 for proteins containing two consecutive arginines in their N terminus. "Y": The protein was predicted by the software.

[b] The number in the TatP column means the score of the corresponding protein.
